# Genome-wide association screens for Achilles tendon and ACL tears and tendinopathy

**DOI:** 10.1371/journal.pone.0170422

**Published:** 2017-03-30

**Authors:** Stuart K. Kim, Thomas R. Roos, Andrew K. Roos, John P. Kleimeyer, Marwa A. Ahmed, Gabrielle T. Goodlin, Michael Fredericson, John P. A. Ioannidis, Andrew L. Avins, Jason L. Dragoo

**Affiliations:** 1 Department Developmental Biology, Stanford University Medical Center, Stanford CA, United States of America; 2 Department Health Research and Policy, Division of Epidemiology, Stanford University Medical Center, Stanford CA, United States of America; 3 Department Orthopaedic Surgery, Stanford University Medical Center, Stanford CA, United States of America; 4 College of Medicine, California Northstate University, Elk Grove CA, United States of America; 5 Department of Medicine, Stanford Prevention Research Center, Stanford University School of Medicine, Stanford CA, United States of America; 6 Department of Health Research and Policy, Division of Epidemiology, Stanford University School of Medicine, Stanford CA, United States of America; 7 Department of Statistics, Stanford University School of Humanities and Sciences, Stanford CA, United States of America; 8 Kaiser Permanente Northern California, Division of Research, Oakland, California, United States of America; University of Pittsburgh, UNITED STATES

## Abstract

Achilles tendinopathy or rupture and anterior cruciate ligament (ACL) rupture are substantial injuries affecting athletes, associated with delayed recovery or inability to return to competition. To identify genetic markers that might be used to predict risk for these injuries, we performed genome-wide association screens for these injuries using data from the Genetic Epidemiology Research on Adult Health and Aging (GERA) cohort consisting of 102,979 individuals. We did not find any single nucleotide polymorphisms (SNPs) associated with either of these injuries with a p-value that was genome-wide significant (p<5x10^-8^). We found, however, four and three polymorphisms with p-values that were borderline significant (p<10^−6^) for Achilles tendon injury and ACL rupture, respectively. We then tested SNPs previously reported to be associated with either Achilles tendon injury or ACL rupture. None showed an association in our cohort with a false discovery rate of less than 5%. We obtained, however, moderate to weak evidence for replication in one case; specifically, rs4919510 in *MIR608* had a p-value of 5.1x10^-3^ for association with Achilles tendon injury, corresponding to a 7% chance of false replication. Finally, we tested 2855 SNPs in 90 candidate genes for musculoskeletal injury, but did not find any that showed a significant association below a false discovery rate of 5%. We provide data containing summary statistics for the entire genome, which will be useful for future genetic studies on these injuries.

## Introduction

Achilles tendinopathy or rupture and anterior cruciate ligament (ACL) ruptures are frequent sources of pain and dysfunction in recreational and elite athletes. Recent studies have tested a few single-nucleotide polymorphisms (SNPs) in a small number of candidate genes for association with Achilles tendon injury or ACL rupture in athletes. For Achilles tendon injury, studies found 19 DNA variations residing in 12 genes that were associated with Achilles tendinopathy at p<0.05 using cohorts containing between 52 and 184 athletes [1–12]. In one case (rs12722 in *COL5A1*), the association with Achilles tendinopathy was found to replicate in an independent cohort (p = .024)[4]. For ACL rupture, studies have previously found nine DNA variations in eight genes with p < .05 where the number of cases ranged between 86 and 358 [7,13, 14–22]. rs180012 in *COL1A1* was replicated in several studies [14–18]. None of the remaining SNPs have been replicated for either Achilles tendon injury or ACL rupture.

The purpose of this study was to perform a screen of the entire genome for polymorphisms associated with either Achilles tendon injury or ACL rupture in a large dataset. We also analyzed specific SNPs previously reported to be associated with either injury.

## Materials and methods

Genome-wide association screens were performed for either Achilles tendon injury (defined as tendinopathy, rupture or repair) or ACL rupture using data from the Genetic Epidemiology Research on Adult Health and Aging (GERA) cohort. The generation of the data and pipeline for data analysis have been previously described in Jorgenson et al., 2015 and Roos et al., 2016 [23] [24].

### GERA cohort

The GERA cohort is comprised of 110,266 adult men and women members of Kaiser Permanente Northern California (KPNC) Medical Care Plan. It is a component of the KPNC Research Program on Genes, Environment and Health (RPGEH). A complete description of the cohort and study design can be found in dbGaP (Study Accession: phs000674.v1.p1). The average age of the participants at the time of sample collection was 62.9 years old (standard deviation = 13.8 years). Length of membership in KPNC averaged 23.5 years. The medical record contains the entire patient history, including injuries that occurred prior to enrollment in KPNC, if reported by the patient and recorded by the physician.

Our analysis cohort (n = 102,979) includes 59,671 females, 43,242 males, and 66 individuals of ambiguous sex. Sex was determined by heterozygosity on the X chromosome [23]. For 66 individuals, heterozygosity of the X chromosome was indeterminate, so no sex call was made. Moreover, our analysis cohort is ethnically diverse, including 83,264 European-White (EUR); 8,560 Latino (LAT); 7,518 East Asian (EAS); 3,161 African-American (AFR); and 476 South Asian (SAS) individuals based on ancestry principle components.

### Genotyping, quality control, imputation, genetic ancestry

Participants were genotyped at over 650,000 SNPs on four ancestry group-specific Affymetrix Axiom genome-wide arrays optimized for individuals of European (EUR), African-American (AFR), East Asian (EAS), and Latino (LAT) ancestry group [25,26]. Genotype quality control procedures for the GERA cohort were performed as described previously, except that only SNPs with a minor allele frequency of >1% were analyzed [27]. The final number of SNPs that were directly genotyped was 670,572 for EUR; 802,186 for LAT; 708,373 for EAS; and 878,176 for AFR arrays.

Genotypes were pre-phased and then imputed to a cosmopolitan reference panel consisting of all individuals from the 1000 Genomes Project [24]. The final number of SNPs that were imputed was 9,207,988 for EUR (9,878,560 total); 10,380,912 for LAT (11,183,098 total); 8,355,578 for EAS (9,063,951 total); and 16,659,640 for AFR arrays (17,537,816 total).

Determination of genetic ancestry was performed by principal component analysis (PCA), as previously described [24]. These ancestry principal components were used in the GWAS to adjust for genetic ancestry.

### Phenotype definition

Achilles tendon injuries were identified in the GERA cohort based on clinical diagnoses and surgical procedures captured in the KPNC Electronic Health Record system. Cases were defined as individuals with at least one International Classification of Disease, Ninth Revision (ICD9) or Common Procedure Terminology, Fourth Edition (CPT4) code: ICD726_71, ICD727_67, and/or CPT27650, describing Achilles bursitis or tendinitis, nontraumatic rupture of Achilles tendon, and primary repair of Achilles tendon rupture, respectively (Table 1). The Electronic Medical Record includes injuries over the entire lifetime of the patients; i.e. those that occurred prior to enrollment in KPNC as well as those that occurred after the genotyping analysis was performed, and until this study was initiated July 22, 2015.

**Table 1:** International Statistical Classification of Diseases and Related Health Problems and Current Procedural Terminology codes for Achilles Tendinopathy.

Description	Code	N (%)^a^
Achilles bursitis or tendinitis	ICD726_71	4,949 (96)
Non-traumatic rupture of Achilles tendon	ICD727_67	105 (2)
Repair, primary, open or percutaneous, ruptured Achilles tendon	CPT27650	215 (4)
Total number individuals		5,148 (100)

^a^Number of individuals with that code in their electronic medical records, and percent of the total number of individuals in parentheses. Some individuals had more than one code.

For ACL rupture, KPNC patients with any potential ACL injury were identified by search of the electronic medical record for the ICD9 codes 717.83 and 844.2 and the CPT4 codes 29888 and 27407 at any time. The imaging studies and surgical reports from these patients were reviewed and those who had strong evidence for a full or partial ACL rupture on MRI and/or underwent ACL reconstruction were considered to have had an ACL rupture. The charts of the remaining patients were then manually reviewed by one of the study physicians (AA) with the assistance of the study orthopedist (JD). Those patients who had confirmatory evidence of ACL rupture (e.g., unambiguous history in a progress note) were also classified as having had an ACL rupture. Patients with ambiguous histories or ACL injury without rupture were not considered to have had a full or partial ACL rupture.

### Genome-wide association and meta-analysis

We tested for SNP associations with a logistic regression model using allele in an additive genetic model for each of the five ancestry groups separately, as in Roos et al., 2016 [24]. The model was adjusted for sex, age at enrollment into the RPGEH cohort, ancestry group using principal components, and genotyping chip version for all populations.

For Achilles tendinopathy or rupture, the principal components used were: AFR (6 PCs), EAS (6 PCs), EUR (10 PCs), LAT (6 PCs) and SAS (6 PCs). For ACL rupture, there were only 7 cases in the SAS and AFR populations, and so these populations were not analyzed further. For the remaining populations for ACL rupture, the principal components used were: EAS (2 PCs), EUR (4 PCs) and LAT (3 PCs). 10,551,193 SNPs were processed in the meta-analysis for Achilles tendinopathy or rupture and 8,303,052 SNPs were processed for ACL rupture meta-analysis.

To assess for inflation due to population stratification, the genomic control parameter (λ) was calculated [28]. For Achilles tendinopathy or rupture, genomic inflation was small for GWA analyses in the EUR, LAT, EAS, AFR populations (λ between .99 and 1.02), but substantial in the SAS population (λ~0.37). For ACL rupture, λ was between .93 and 1.0 for the EAS, EUR and LAT populations. Subsequently, p-values were adjusted for genomic control in each population. The linkage disequilibrium score method was used to evaluate the genomic inflation factor using the python script ldsc from Bulik-Sullivan et al. 2015 [29]. LD Hub was used to compute the genetic correlation between the GWAS for the two injuries from this paper and those from 198 published studies (http://ldsc.broadinstitute.org/)[30]. The input data contained the unadjusted p-values from the European populations from either the Achilles tendon injury or the ACL injury GWAS. SNPs that overlapped those in HAPMAP3 were retained for analysis. For both the Achilles and ACL injury phenotypes, there was a low intercept of the LD score regression, indicating lack of bias from population structure (S1 Table). Results from each population were combined by inverse-variance, fixed-effects meta-analysis and by inverse-variance, random-effects meta-analysis. Results are reported using fixed-effects meta-analysis p-values. SNPs were discarded from the meta-analysis if data were missing from the EUR population, which comprises over 80% of the cohort.

The fixed-effects and random-effects p-values for all of the SNPs tested in this study are available from NIH GRASP: https://grasp.nhlbi.nih.gov/FullResults.aspx. These data could be useful for future studies on the genetic risk for Achilles tendon injury or ACL rupture.

### Replication analysis of previously reported SNPs

We searched our meta-analysis results for previously-published genetic associations with either Achilles injury or ACL rupture [1,2,4–12,13, 14–22]. We searched the public databases PubMed/MEDLINE (http://www.ncbi.nlm.nih.gov/pubmed) and Scopus (http://www.scopus.com/) for previously published studies on the genetics of Achilles tendon injury or ACL rupture. Inclusion criteria for filtering results were: research papers with original data, English language, full-text article available (or abstract with enough data), genetic association studies with human subjects, cases with Achilles tendon injury or ACL rupture phenotypes, and gene or SNP associations reported. Articles were included regardless of whether or not the associations were significant and regardless of the size of the study population. Furthermore, the references from each publication were used to find other articles not captured in the initial search. The final searches were conducted on June 1, 2016. A Benjamini-Hochberg false discovery rate threshold (q = 0.05) was used to account for multiple testing [31].

### Analysis of novel candidate SNPs

Based on our knowledge of biological functions of the Achilles tendon and ACL, we generated a list of 90 candidate genes potentially associated with either Achilles or ACL injury. These genes code for structural components or the development of ligaments and/or tendons. A set of all known SNPs found within 2kb of the start and end of these genes was generated using SCAN (http://www.scandb.org/) [31]. Rare SNPs (MAF<0.005) were removed because they would not have enough statistical power to show an association in gene association studies. SNPs not found in our GWAS results were removed, yielding 14,734 SNPs. To select a single tag SNP in each linkage disequilibrium block, SNP pruning with an LD threshold (r^2^>0.5) was performed in PLINK v1.90 (b3.34), resulting in 2,855 candidate SNPs. For the candidate genes that had been tested earlier, previous studies tested only one or a few SNPs from each gene whereas our list contains many tag SNPs spanning the entire gene. Because we tested many candidate SNPs, we used the Benjamini-Hochberg method to set the false discovery rate to q = .05; i.e. the top SNP in the list would require a p-value of 1.7x10^-5^ in order to be deemed significant [32].

Gene-based testing was performed using the VErsatile Gene-bASed test VEGAS2 [33]. 2855 SNPs from 90 candidate genes and their fixed-effects p-values from either the ACL rupture or the Achilles tendon injury meta-analyses were used. DNA variants that were not SNPs were filtered from the input files. Gene-based analysis was performed using the online VEGAS2 software from https://vegas2.qimrberghofer.edu.au/ [33].

### Ethical considerations

This study analyzed stored data from RPGEH. The health and genotype data for the subjects were de-identified. All study procedures were approved by the Institutional Review Board of the Kaiser Foundation Research Institute.

## Results

### Study population and genotype information

We obtained access to injury information and genotype data from the Research Program on Genes, Environment and Health cohort (Materials and Methods). This program includes genotype and medical data from 102,979 patients in the Kaiser-Permanente system in Northern California. For Achilles tendon injury, we interrogated the electronic medical records of these individuals for those that had incurred tendinopathy or a rupture (Table 1)(Materials and Methods). For ACL rupture, cases were defined as patients that showed ACL rupture by MRI or that had undergone a procedure for ACL reconstruction (Methods). In total, there were 5,148 cases of Achilles tendon injury (consisting of 290 with ruptures and 4,858 with bursitis or tendinitis) from 102,979 individuals. For ACL rupture, there were only 7 cases each from the AFR and SAS populations, so these ancestry groups were excluded from further analysis. This left 598 cases of ACL rupture from 99,342 individuals in the EAS, EUR and LAT ancestry groups. Descriptive factors for these injuries are shown in Table 2. For ACL rupture, the average age of the cases is 10 years less than that of the controls (p<10^−100^). One possible explanation is that there could be an ascertainment bias against ACL ruptures in older patients that incurred the injury when they were young before MRI was commonly used.

**Table 2:** Descriptive factors for Achilles tendon injury and ACL rupture.

**Achilles tendon injury**			
	Cases (%)	Controls (%)	Total
Overall	5,148 (5.0%)	97,831 (95.0%)	102,979
**Ancestry Group**			
European	4,258 (5.1%)	79,006 (94.9%)	83,264
Latin-American	413 (4.8%)	8,147 (95.2%)	8,560
East Asian	268 (3.6%)	7,250 (96.4%)	7,518
African	192 (6.1%)	2,969 (93.9%)	3,161
South-East Asian	17 (3.6%)	459 (96.4%)	476
**Sex**			
Female	2,934 (4.9%)	56,737 (95.1%)	59,671
Male	2,211 (5.1%)	41,031 (94.9%)	43,242
Unknown	3 (4.5%)	63 (95.5%)	66
Age^a^	62.3 (62.2–62.4)^b^	62.7 (62.6–62.8)	62.6 (62.5–62.7)
**ACL rupture**			
	Cases (%)	Controls (%)	Total
Overall	598 (.61%)	98,744 (99.39%)	99,342
**Ancestry Group**			
European	495 (.59%)	82,769 (99.61%)	83,264
Latin-American	54 (.63%)	8,506 (99.37%)	8,560
East Asian	49 (.65%)	7,469 (99.35%)	7,518
**Sex**			
Female	349 (.61%)	57,257 (99.39%)	57,606
Male	249 (.59%)	41,421 (99.41%)	41,670
Unknown	0 (0%)	66 (100%)	66
Age^a^	52.0 (50.8–53.1)^c^	62.7 (62.6–62.8)	62.6 (62.5–62.7)

^a^ Mean age (years) at enrollment (95% CI).

^b^There is a small difference in ages between cases and controls (p = 0.01).

^c^ The difference in ages between the cases and controls is highly significant (p<10^−100^).

### Genome-wide studies for association with either achilles tendon Injury or ACL rupture

**Fig 1:**
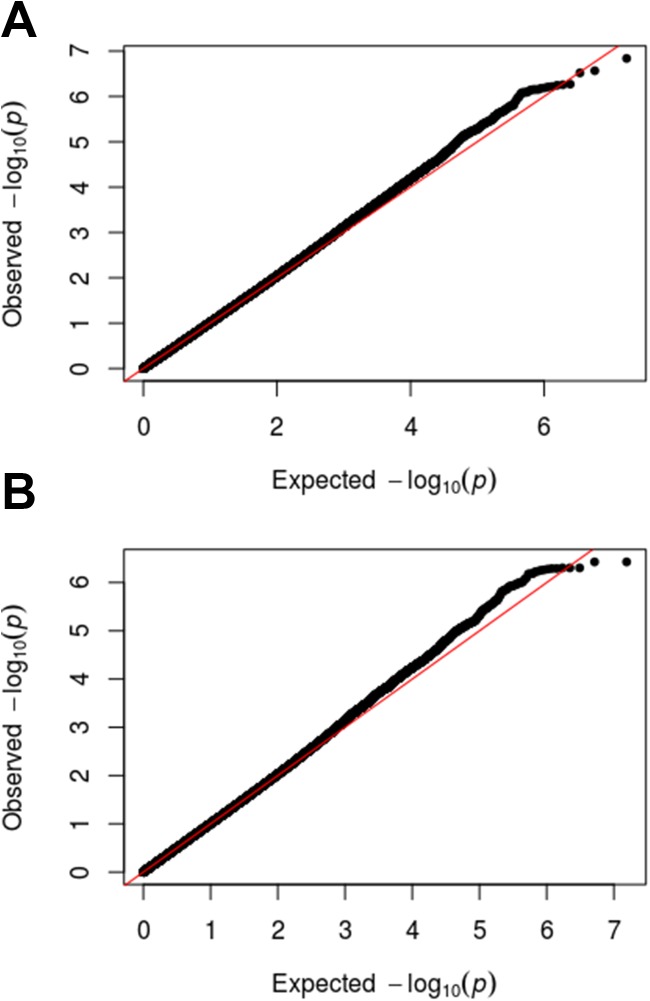
**QQ plots for Achilles tendon injury (A) or ACL rupture (B).** The plots show the observed p-values plotted on the y-axis (black dots) against the p-values expected by chance plotted on the x-axis (red line). The black dots align closely with the red line indicating that there is little or no statistical signal in either the Achilles tendon injury or ACL rupture cohorts.

Fig 1 shows QQ plots with the observed p-values from the GERA cohort compared to the p-values that would be expected by chance. For either injury, the p-values from the association study roughly overlap those that would be expected by chance.

We used Linkage Disequilibrium Score Regression to analyze the GWAS data for: 1) heritability, 2) fraction of the genetic association signal derived from polygenic associations, and 3) overlap with GWAS data for other traits [29,30]. First, genetic heritability refers to amount of the trait that can be explained by the sum of all of the SNPs in the GWAS. For both Achilles tendon injury and ACL rupture, the heritability was low (0.5–1.1%)(S1 Table). Second, LD score regression is able to discern whether the observed genetic associations are due to associations with small effects from many loci versus associations due to population structure. If the associations are due to polygenic associations, then the intercept from LD score regression will be less than the genomic inflation factor. For both Achilles tendon injury and ACL rupture, LD score regression suggests that population structure did not have a strong influence in the observed association results. Third, the amount of genetic correlation between the results from the two studies analyzed here with those from 198 previously published studies was determined using LD Hub (Materials and Methods)[30]. Neither of the GWAS results from Achilles tendon injury or ACL rupture showed a significant genetic correlation with any of the 198 datasets (S2 Table).

**Fig 2:**
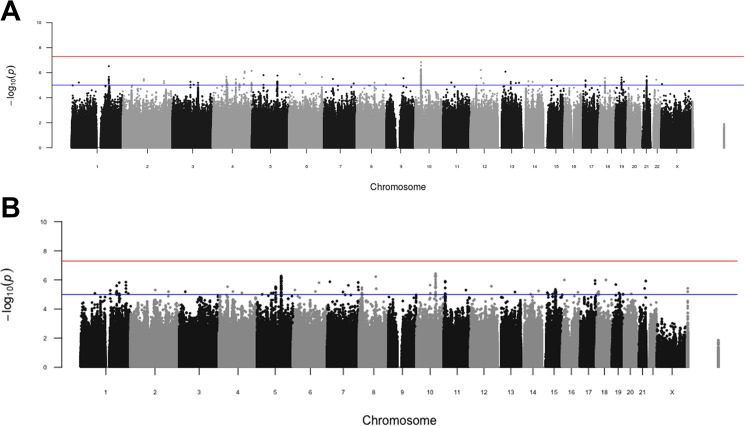
**Manhattan plots for Achilles tendon injury (A) or ACL rupture (B)**. Shown are the p-values for association with Achilles tendon injury or ACL rupture. Chromosome number is indicated across the bottom. Y-axes show the -log_10_ p-value for association with either Achilles tendon injury or ACL rupture. The red line indicates the threshold for genome-wide significance (p = 5x10^-8^) and the blue line indicates p = 5x10^-5^.

We plotted the p-value for every SNP on Manhattan plots (Fig 2). We did not find any SNP that matched the genome-wide statistical threshold of p<5x10^-8^. Table 3 lists the independent SNPs that reach p<10^−6^. There were four SNPs for Achilles tendon injury and three SNPs for ACL rupture.

**Table 3:** Top hits from the genome-wide screen for Achilles tendon injury and ACL rupture (p<1x10^-6^).

Achilles tendon injury						
SNP	EA^a^	MAF^b^	P (FE)^c^	OR (FE)(95% CI)^d^	P (RE)	OR (RE) (95% CI)
rs1937810	C	0.17	1.5x10^-7^	1.16 (1.11–1.21)	1.5x10^-7^	1.16 (1.11–1.21)
rs57104447	C	0.04	3.0x10^-7^	1.28 (1.19–1.37)	.01	1.25 (1.16–1.34)
rs57224706	G	0.03	6.0x10^-7^	1.36 (1.24–1.48)	6.0x10^-7^	1.36 (1.24–1.48)
rs60713544	C	0.02	7.2x10^-7^	1.43 (1.29–1.57)	7.2x10^-7^	1.43 (1.29–1.57)
ACL rupture						
SNP	EA^a^	MAF^b^	P (FE)^c^	OR (FE) (95% CI)^d^	P (RE)	OR (RE) (95% CI)
rs4067493	G	0.04	2.4x10^-7^	1.94(1.69–2.19)	3.1x10^-5^	2.06(1.81–2.31)
rs113435565	C	0.03	4.0x10^-7^	1.91 (1.66–2.16)	4.0x10^-7^	1.91 (1.66–2.16)
rs11960097	G	0.08	5.9x10^-7^	1.54 (1.37–1.71)	5.9x10^-7^	1.54 (1.37–1.71)

^a^Effect allele.

^b^Minor Allele Frequency in the control population.

^c^P-value from fixed effects (FE) or random effects (RE) meta-analysis.

^d^Allelic odds ratio (95% confidence interval) for the effect allele. FE, fixed effect; RE, random effect.

### Re-testing candidate SNPs for association with achilles tendon Injury or ACL rupture

Previous studies have reported candidate SNPs that have shown an association with either Achilles tendon injury or ACL rupture using p<0.05 as a cutoff. Specifically, 19 SNPs in 12 candidate genes have been reported to show an association with Achilles tendinopathy (using p<0.05 as a cutoff)[1,2,4–12]. Likewise, nine SNPs in eight candidate genes have been reported to show an association with ACL rupture [7,13,14–22].

For Achilles tendinopathy or rupture, 14 of these SNPs were contained in our dataset whereas for ACL rupture, 6 SNPs were present. We attempted to replicate the previous results for these candidate SNPs using our dataset. Because we are testing only a small number of SNPs, the threshold for statistical significance can be much lower than the genome-wide threshold that we used for the genome-wide study above (p<5x10^-8^). It is still important, however, to adjust the p-value threshold to compensate for multiple testing. We used the Benjamini-Hochberg method to set the false discovery rate to q = 0.05. In addition, the association should show the same direction of effect (i.e. same risk allele) as in the previous publication.

For Achilles tendinopathy or rupture, none of the 14 SNPs were found to be significant using the Benjamini-Hochberg threshold (Table 4). However, rs1045485 and rs4789932 showed small p-values of p<0.01, but in both cases the direction of the effect in our study was opposite to the direction reported previously (Table 4). For ACL rupture, none of the SNPs showed an association in our data set using the Benamini-Hochberg cutoff (Table 4).

**Table 4:** Re-testing candidate genes for association with Achilles tendon injury or ACL rupture.

**Achilles tendon injury**					
**SNP**	**Gene**	**EA**^a^	**P-value**^b^	**OR (95% CI)**	**Ref**^c^
rs1045485^d^	CASP8	C	2.6E-03	1.10 (1.04–1.16)	[2]
rs4747096	ADAMTS14	G	0.55	0.98 (0.93–1.04)	[10]
rs2761884	BMP4	T	0.22	1.03 (0.98–1.07)	[1]
rs1134170	COL5A1	A	0.45	0.97 (0.88–1.05)	[5]
rs12722	COL5A1	T	0.62	0.97 (0.88–1.06)	[4]
rs3196378	COL5A1	A	0.75	1.01 (0.93–1.10)	[4]
rs1559186	COL5A3	C	0.75	1.01 (0.97–1.05)	[6]
rs331079	FBN2	C	0.32	1.03 (0.93–1.10)	[7]
rs4919510	MIR608	G	0.35	0.98 (0.92–1.03)	[5]
rs591058	MMP3	T	0.34	1.06 (1.02–1.10)	[9]
rs679620	MMP3	T	0.35	1.06 (1.02–1.10)	[9]
rs4789932^d^	TIMP2	G	8.7E-03	1.08 (1.02–1.13)	[10]
rs1330363	TNC	C	0.26	1.02 (.98–1.07)	[12]
rs2104772	TNC	A	0.29	0.98 (.94–1.02)	[12]
**ACL rupture**					
**SNP**	**Gene**	**EA**^a^	**P-value**^b^	**OR (95% CI)**	**Ref**^c^
rs1516797	ACAN	T	0.99	1.00 (0.77–1.43)	[13]
rs516115	DCN	A	0.29	1.07 (0.94–1.18)	[13]
rs970547	COL12A1	T	0.11	1.11 (0.98–1.25)	[19]
rs2276109	MMP12	T	0.44	1.07 (0.90-.1.24)	[20]
rs1800255	COL3A1	A	0.17	1.09 (1.00–1.31)	[21]
rs331079	FBN2	G	0.45	1.07 (.91–1.25)	[7]

^a^ Effect Allele.

^b^ P-value from this study.

^c^ Reference showing original association of candidate SNP with Achilles tendinopathy.

^d^For rs1045485 and rs4789932, the direction of the effect was opposite to the published results; i.e. the minor C allele of rs1045485 and the G allele of rs4789932 are associated with increased risk in this work but decreased risk in the prior work [2][10].

In our gene association study, we used sex, age and ancestry group as covariates whereas previous studies did not use these covariates. To control for this difference, we re-tested the candidate SNPs without using covariates in order to align our analysis with those that had been done previously (S3 Table). For Achilles tendinopathy or rupture, one SNP in *MIR608* (rs4919510) showed a low p-value (p = 5.1x10^-3^) and had the same direction of effect (G is protective) as seen previously [5]. The p-value for rs4919510 (p = 5.1x10^-3^) is close to the p-value threshold set using the Benjamini-Hochberg method (p = 3.5x10^-3^); i.e. a p-value of 5.1x10^-3^ has about a 7% chance to be a false replication using 14 tests. Additionally, four other SNPs (rs1045485, rs591058, rs679620 and rs4789932) showed a small p-value but their direction of effect was opposite to the effect seen previously, so these SNPs do not provide support for the previous findings (S3 Table).

For ACL rupture, when we repeated the analysis without using covariates, we found that rs1800255 in *COL3A1* had a small p-value of 0.03 with the same direction as found previously (S3 Table)[21]. With seven tests, this p-value does not meet the Benjamini-Hochberg threshold (p = 7x10^-3^) and has about a 21% chance of being a false discovery.

### Testing new candidate SNPs for association with achilles tendon Injury or ACL rupture

To demonstrate how these genome-wide data can be applied, we used the data to evaluate a new set of candidate genes for association with Achilles tendinopathy or rupture and with ACL rupture. We created a list of 90 genes encoding proteins involved in the formation of ligaments or tendons, including the set of candidate genes from previous publications (S4 Table). We assembled a list of all independent, common SNPs in these 90 genes. We tested each of the candidate SNPs for association with these injuries by looking up their p-values in the meta-analysis results (S5 Table). None of the candidate SNPs had a significant p-value for either injury (using the Benjamini-Hochberg false discovery rate of q = 0.05). The top SNP for Achilles tendon injury (rs4660148) had p = 5x10^-5^, which corresponds to a false discovery rate of about 14%. The top SNP for ACL rupture (rs8090) had p = 6x10^-4^, which corresponds to a false discovery rate greater than 50% (S5 Table).

Besides evaluating each SNP individually, SNPs were analyzed as part of genes using gene-based analysis [33]. In gene-based analysis, SNPs are assigned to individual genes in close proximity, and then each gene is tested for association with a phenotype. Gene-based testing confers an advantage in cases where there is allelic heterogeneity–when there are multiple, independent variants influencing a trait at the same locus. With gene-based testing, the signals for genetic association from independent variants are combined, possibly yielding a p-value below a given significance threshold even though the individual SNPs in the gene may not themselves have significant p-values. We used gene-based analysis to test the 90 candidate genes for association with either Achilles tendinopathy or rupture or with ACL rupture (Methods). For Achilles tendinopathy or rupture, none of the genes showed a significant association (S6 Table).

For ACL rupture, *GLT25D1* (also referred to as *COLGALT1*) initially showed a borderline significant association using gene-based analysis. Specifically, *GLT25D1* had a p-value of 5.7x10^-4^ among 87 genes tested, which is just below the threshold for Bonferroni significance (p = 5.8x10^-4^)(S6 Table). *GLT25D1* encodes collagen beta(1-O)galactosyltransferase 1, which transfers galactose to hydroxylysine residues of collagen [34]. Four SNPs (rs2082001, rs2375637, rs8110571 and rs55960725) within *GLT25D1* showed an association with ACL rupture with p<0.05 (S1 Fig, S7 Table). One possibility is that each of these SNPs might influence ACL rupture independently (allelic heterogeneity; four independent alleles). Another possibility is that only one SNP is responsible for the association and the other SNPs may show an association due to weak linkage (r^2^ was between 0.03 and 0.43 between the four SNPs). To distinguish between these two possibilities, we performed a conditional analysis in which the genotype of the SNP with the strongest p-value (rs55960725) was added as a covariate in the logistic regression for ACL rupture. In the conditional analysis, none of the other SNPs (rs2082001, rs2375637 or rs8110571) had p-values that fell below 0.05 and their effect sizes shrank, indicating that their association with ACL rupture was partially dependent on rs55960725 (S7 Table).

## Discussion

Achilles tendon and ACL injury are common in recreational and elite athletes, and even in non-athletes [35–40]. Prior studies have identified 12 genes associated with Achilles tendinopathy and 8 genes associated with ACL rupture [1,2,4–12,13, 14–22]. These prior studies, however, evaluated a small number of candidate genes among small cohorts of athletes. With the advent of large-scale genotyping programs, it is now possible to screen the entire genome for polymorphisms associated with sports injury risks such as Achilles tendon or ACL injury. In principle, a genome-wide screen for injury could provide new insight about the differences between individuals regarding their inherent propensity for injury. Furthermore, because the genotype data includes most common polymorphisms, a genome-wide screen reports the strongest associations in the genome without bias, and these associations are the most useful for predicting individual risks for injury.

Here, we performed a study to find genetic variants associated with Achilles tendon or ACL injury by obtaining access to large-scale genotype and phenotype data from the Research Program on Genes, Environment and Health. The data contains information from 102,979 individuals of whom 5,418 had Achilles tendinopathy or rupture (from all five ancestry groups) and 598 had an ACL rupture (from the EAS, EUR and LAT ancestry groups). To date, this is the largest gene association study for either Achilles tendon or ACL injury reported in terms of number of SNPs that were genotyped and number of injury cases.

We were unable to find any SNPs associated with either injury type at a genome-wide significance level. For a hypothetical common SNP with a minor allele frequency of 5% and a genotype relative risk of 1.3, power calculations indicate that this SNP would have about a 78% chance of being detected in our Achilles study and a 59% chance for detection in our ACL study. Thus, it is unlikely that there are many common SNPs with a medium-to-strong association with these injuries (i.e. genotype relative risk > 1.3).

We found four independent polymorphisms, however, associated with Achilles tendinopathy or rupture with a borderline significant p-value between 1x10^-6^ and 5x10^-8^ (Table 3). Similarly, three independent polymorphisms were associated with ACL rupture with borderline significance (Table 3). A previous analysis tallied available SNP associations with borderline associations (between p≤10^−7^ and p>5x10^-8^), and found that 73% were replicated in subsequent studies [41]. For p-values between 10^−6^ and 10^−7^ the replication rate is likely to be much smaller, but not negligible [42]. Thus, some of the SNPs presented in Table 3 may be replicated in future studies.

Previous candidate gene studies have reported some SNPs that show a weak association with Achilles tendon or ACL injury [1,2,4–12,13,14–22]. To identify weak SNPs like these candidates in a genome-wide screen, it will be necessary to either increase the size of the study (screen a much larger cohort than the ones used here) or to improve the precision in the diagnoses of the injuries.

Although we re-tested candidate SNPs that were previously reported to show an association with either Achilles or ACL injuries, we did not find strong evidence for replication of the candidate gene results. We performed the replication analysis using age, sex and ancestry group as covariates (as was done in the genome-wide analysis) as well as without these covariates (as was done in the candidate gene studies). For Achilles tendinopathy or rupture, we found moderate evidence for replication of one of the 14 tested SNPs; specifically, rs4919510 in *MIR608* had a p-value of 5.1x10^-3^ without using covariates that suggests a false discovery rate of about 7%, which is almost statistically significant (S3 Table). None of the other SNPs showed evidence for replication either with or without using covariates in the analysis.

For ACL rupture, none of the other SNPs showed evidence of replication either with or without using covariates in the analysis. Previous work has shown that the association of rs12722 in *COL5A1* with Achilles tendinopathy could be replicated [3,4,14–18]. However, this SNP did not show a significant association with Achiles tendinopathy or rupture in our study (p = 0.62). Evidence from many other genetic association studies suggests that candidate gene associations need to be independently replicated, otherwise their credibility is low [43,44]. For Achilles tendinopathy or rupture, our study included 5,418 cases compared to between 52 and 184 cases in previous studies [1,2,4–12]. For ACL rupture, our study had 598 cases compared to between 86 and 358 cases used previously [7,13, 14–22]. The larger number of cases in our study compared to previous ones indicates that we have good statistical power to replicate the previously-reported associations.

One possible explanation for the failure to replicate previous candidate gene studies is that our cohort consists of patients in the Kaiser-Permanente medical system in Northern California regardless of activity level, whereas previous studies evaluated cohorts of competitive athletes. Varying levels of physical activity may affect risk for sustaining an Achilles tendon injury or ACL rupture.

For Achilles tendon injury, another possible explanation for the failure to replicate previous results is that cases were identified based on diagnosis and procedure codes in the electronic medical record. Patients in large, administrative data sets may have been misdiagnosed, introducing information bias. Non-differential misclassification of Achilles tendon injury would tend to reduce the strength of associations. A second possible explanation is that we evaluated patients with Achilles tendinopathy, bursitis or rupture as a single injury group. Achilles tendon rupture may represent an increased injury severity versus tendinopathy with a correspondingly stronger genetic effect, or conversely may be associated with acute trauma with limited genetic effect. A third possibility is that the genetic risk factors for Achilles bursitis may be different from those for intrinsic Achilles tendon pathology. Identifying cases of ACL rupture was more definitive, as cases had either shown an ACL rupture by MRI or had undergone an ACL reconstruction procedure.

We performed a candidate-gene study for Achilles tendon injury and ACL rupture using 2855 SNPs in 90 candidate genes. In the first analysis, SNPs were tested individually but none showed a significant association with either type of injury. In the second analysis, gene-based analysis was used to aggregate SNPs into genes and then test each of the genes for association with these injuries. None of the genes showed an association with Achilles tendon injury but *GLT25D1* (which encodes a protein that glycosylates collagen) showed a borderline significant association with ACL rupture. *GLT25D1* contained four SNPs with nominally significant p-values. However, it is unclear if the gene-based result for *GLT25D1* is due to multiple, independent SNPs contributing to risk for ACL rupture or due to weak linkage between SNPs in the gene.

The summary statistics from the GWAS for Achilles tendon and ACL injury are available to the public at NIH GRASP: https://grasp.nhlbi.nih.gov/FullResults.aspx. These tables may be used in future genetic studies. For instance, one could make a list of additional genes and genetic pathways involved in the development, formation or structure of tendons/ligaments, and then use the data presented here to test those genes for an association. In addition, the data from this paper could be used to validate SNPs found in a first-stage GWAS for Achilles tendon or ACL injury. Finally, these data could be combined with data from other musculoskeletal injuries (e.g. rotator cuff injuries) in a cross-phenotype meta-analysis in order to find SNPs associated with musculoskeletal injuries in general.

## Supporting information

S1 Fig Regional-association plot for *GLT25D1*. Tested SNPs within *GLT25D1* are arranged by genomic position on chromosome 19 (x-axis). The y-axis indicates -log_10_ p-values for association with ACL rupture for each SNP. The four SNPs showing nominal statistical significance (p<0.05) are shown. The color of dots representing flanking SNPs indicates their linkage disequilibrium (r^2^) with the lead SNP as indicated in the heat map color key.
(TIF)

S1 Table Results from LD Score Regression. (DOCX)

S2 Table Genetic correlation results from LD Hub for Achilles tendon injury and ACL rupture. A. Achilles tendon injury. B. ACL rupture.
(XLS)

S3 Table Re-testing candidate genes for association with Achilles tendon injury or ACL rupture without using co-variates. (DOCX)

S4 Table List of 90 Candidate Genes. (DOCX)

S5 Table Summary statistics for 2855 SNPs in 90 candidate genes for association with Achilles tendinopathy/rupture or ACL rupture. (XLSX)

S6 Table Gene-based analysis of 90 candidate genes for association with Achilles tendon injury or ACL rupture. (XLS)

S7 Table Association of SNPs in *GLT25D1* with ACL rupture. (DOCX)

## References

[1] Salles JI, Amaral MV, Aguiar DP, Lira DA, Quinelato V, Bonato LL, et al BMP4 and FGF3 haplotypes increase the risk of tendinopathy in volleyball athletes. Journal of science and medicine in sport / Sports Medicine Australia. 2015;18(2):150–5. 10.1016/j.jsams.2014.02.011 24661680

[2] Nell EM, van der Merwe L, Cook J, Handley CJ, Collins M, September AV. The apoptosis pathway and the genetic predisposition to Achilles tendinopathy. Journal of orthopaedic research: official publication of the Orthopaedic Research Society. 2012;30(11):1719–24. 22588838 10.1002/jor.22144

[3] Mokone GG, Schwellnus MP, Noakes TD, Collins M. The COL5A1 gene and Achilles tendon pathology. Scandinavian journal of medicine & science in sports. 2006;16(1):19–26. 16430677 10.1111/j.1600-0838.2005.00439.x

[4] September AV, Cook J, Handley CJ, van der Merwe L, Schwellnus MP, Collins M. Variants within the COL5A1 gene are associated with Achilles tendinopathy in two populations. British journal of sports medicine. 2009;43(5):357–65. 10.1136/bjsm.2008.048793 18443036

[5] Abrahams Y, Laguette MJ, Prince S, Collins M. Polymorphisms within the COL5A1 3'-UTR that alters mRNA structure and the MIR608 gene are associated with Achilles tendinopathy. Annals of human genetics. 2013;77(3):204–14. 10.1111/ahg.12013 23347277

[6] Saunders CJ, van der Merwe L, Cook J, Handley CJ, Collins M, September AV. Extracellular matrix proteins interact with cell-signaling pathways in modifying risk of achilles tendinopathy. Journal of orthopaedic research: official publication of the Orthopaedic Research Society. 2015;33(6):898–903. Epub 2015/02/03. 25640225 10.1002/jor.22820

[7] Khoury LE, Posthumus M, Collins M, van der Merwe W, Handley C, Cook J, et al ELN and FBN2 gene variants as risk factors for two sports-related musculoskeletal injuries. International journal of sports medicine. 2015;36(4):333–7. 10.1055/s-0034-1390492 25429546

[8] Posthumus M, Collins M, Cook J, Handley CJ, Ribbans WJ, Smith RK, et al Components of the transforming growth factor-beta family and the pathogenesis of human Achilles tendon pathology—a genetic association study. Rheumatology. 2010;49(11):2090–7. 10.1093/rheumatology/keq072 20360039

[9] Raleigh SM, van der Merwe L, Ribbans WJ, Smith RK, Schwellnus MP, Collins M. Variants within the MMP3 gene are associated with Achilles tendinopathy: possible interaction with the COL5A1 gene. British journal of sports medicine. 2009;43(7):514–20. 10.1136/bjsm.2008.053892 19042922

[10] El Khoury L, Posthumus M, Collins M, Handley CJ, Cook J, Raleigh SM. Polymorphic variation within the ADAMTS2, ADAMTS14, ADAMTS5, ADAM12 and TIMP2 genes and the risk of Achilles tendon pathology: a genetic association study. Journal of science and medicine in sport / Sports Medicine Australia. 2013;16(6):493–8. 10.1016/j.jsams.2013.02.006 23491141

[11] Mokone GG, Gajjar M, September AV, Schwellnus MP, Greenberg J, Noakes TD, et al The guanine-thymine dinucleotide repeat polymorphism within the tenascin-C gene is associated with achilles tendon injuries. The American journal of sports medicine. 2005;33(7):1016–21. 10.1177/0363546504271986 15983124

[12] Saunders CJ, van der Merwe L, Posthumus M, Cook J, Handley CJ, Collins M, et al Investigation of variants within the COL27A1 and TNC genes and Achilles tendinopathy in two populations. Journal of orthopaedic research: official publication of the Orthopaedic Research Society. 2013;31(4):632–7. 23192621 10.1002/jor.22278

[13] Mannion S, Mtintsilana A, Posthumus M, van der Merwe W, Hobbs H, Collins M, et al Genes encoding proteoglycans are associated with the risk of anterior cruciate ligament ruptures. British journal of sports medicine. 2014;48(22):1640–6. Epub 2014/02/21. 10.1136/bjsports-2013-093201 24552666

[14] Khoschnau S, Melhus H, Jacobson A, Rahme H, Bengtsson H, Ribom E, et al Type I collagen alpha1 Sp1 polymorphism and the risk of cruciate ligament ruptures or shoulder dislocations. The American journal of sports medicine. 2008;36(12):2432–6. Epub 2008/08/02. 10.1177/0363546508320805 18669982

[15] Posthumus M, September AV, Keegan M, O'Cuinneagain D, Van der Merwe W, Schwellnus MP, et al Genetic risk factors for anterior cruciate ligament ruptures: COL1A1 gene variant. British journal of sports medicine. 2009;43(5):352–6. Epub 2009/02/06. 10.1136/bjsm.2008.056150 19193663

[16] Ficek K, Cieszczyk P, Kaczmarczyk M, Maciejewska-Karlowska A, Sawczuk M, Cholewinski J, et al Gene variants within the COL1A1 gene are associated with reduced anterior cruciate ligament injury in professional soccer players. Journal of science and medicine in sport / Sports Medicine Australia. 2013;16(5):396–400. Epub 2012/11/22. 10.1016/j.jsams.2012.10.004 23168334

[17] Posthumus M, September AV, Schwellnus MP, Collins M. Investigation of the Sp1-binding site polymorphism within the COL1A1 gene in participants with Achilles tendon injuries and controls. Journal of science and medicine in sport / Sports Medicine Australia. 2009;12(1):184–9. Epub 2008/03/21. 10.1016/j.jsams.2007.12.006 18353721

[18] Stepien-Slodkowska M, Ficek K, Eider J, Leonska-Duniec A, Maciejewska-Karlowska A, Sawczuk M, et al The +1245g/t polymorphisms in the collagen type I alpha 1 (col1a1) gene in polish skiers with anterior cruciate ligament injury. Biology of sport. 2013;30(1):57–60. Epub 2014/04/20. PubMed Central PMCID: PMCPMC3944561. 10.5604/20831862.1029823 24744467 PMC3944561

[19] Posthumus M, September AV, O'Cuinneagain D, van der Merwe W, Schwellnus MP, Collins M. The association between the COL12A1 gene and anterior cruciate ligament ruptures. British journal of sports medicine. 2010;44(16):1160–5. Epub 2009/05/16. 10.1136/bjsm.2009.060756 19443461

[20] Posthumus M, Collins M, van der Merwe L, O'Cuinneagain D, van der Merwe W, Ribbans WJ, et al Matrix metalloproteinase genes on chromosome 11q22 and the risk of anterior cruciate ligament (ACL) rupture. Scandinavian journal of medicine & science in sports. 2012;22(4):523–33. Epub 2011/03/18. 21410539 10.1111/j.1600-0838.2010.01270.x

[21] Stepien-Slodkowska M, Ficek K, Maciejewska-Karlowska A, Sawczuk M, Zietek P, Krol P, et al Overrepresentation of the COL3A1 AA genotype in Polish skiers with anterior cruciate ligament injury. Biology of sport. 2015;32(2):143–7. Epub 2015/06/11. PubMed Central PMCID: PMCPMC4447760. 10.5604/20831862.1144416 26060338 PMC4447760

[22] Malila S, Yuktanandana P, Saowaprut S, Jiamjarasrangsi W, Honsawek S. Association between matrix metalloproteinase-3 polymorphism and anterior cruciate ligament ruptures. Genetics and molecular research: GMR. 2011;10(4):4158–65. Epub 2011/11/08. 10.4238/2011.October.31.1 22057989

[23] Jorgenson E, Makki N, Shen L, Chen DC, Tian C, Eckalbar WL, et al A genome-wide association study identifies four novel susceptibility loci underlying inguinal hernia. Nature communications. 2015;6:10130 PubMed Central PMCID: PMC4703831. 10.1038/ncomms10130 26686553 PMC4703831

[24] Roos TR, Roos AK, Avins AL, Ahmed MA, Kleimeyer JP, Frederidson M, et al. Genome-Wide Association Study Identifies a Locus Near Cadherin8 Associated With Rotator Cuff Injury. Am J Sports Med. submitted. 10.1371/journal.pone.0189317 PMC5724859 29228018

[25] Hoffmann TJ, Kvale MN, Hesselson SE, Zhan Y, Aquino C, Cao Y, et al Next generation genome-wide association tool: design and coverage of a high-throughput European-optimized SNP array. Genomics. 2011;98(2):79–89. PubMed Central PMCID: PMC3146553. 10.1016/j.ygeno.2011.04.005 21565264 PMC3146553

[26] Hoffmann TJ, Zhan Y, Kvale MN, Hesselson SE, Gollub J, Iribarren C, et al Design and coverage of high throughput genotyping arrays optimized for individuals of East Asian, African American, and Latino race/ethnicity using imputation and a novel hybrid SNP selection algorithm. Genomics. 2011;98(6):422–30. PubMed Central PMCID: PMC3502750. 10.1016/j.ygeno.2011.08.007 21903159 PMC3502750

[27] Kvale MN, Hesselson S, Hoffmann TJ, Cao Y, Chan D, Connell S, et al Genotyping Informatics and Quality Control for 100,000 Subjects in the Genetic Epidemiology Research on Adult Health and Aging (GERA) Cohort. Genetics. 2015;200(4):1051–60. PubMed Central PMCID: PMC4574249. 10.1534/genetics.115.178905 26092718 PMC4574249

[28] R. Team. R: A language and environment for statistical computing2013.

[29] Bulik-Sullivan BK, Loh PR, Finucane HK, Ripke S, Yang J, Schizophrenia Working Group of the Psychiatric Genomics C, et al LD Score regression distinguishes confounding from polygenicity in genome-wide association studies. Nature genetics. 2015;47(3):291–5. PubMed Central PMCID: PMC4495769. 10.1038/ng.3211 25642630 PMC4495769

[30] Bulik-Sullivan B, Finucane HK, Anttila V, Gusev A, Day FR, Loh PR, et al An atlas of genetic correlations across human diseases and traits. Nature genetics. 2015;47(11):1236–41. PubMed Central PMCID: PMC4797329. 10.1038/ng.3406 26414676 PMC4797329

[31] Gamazon ER, Zhang W, Konkashbaev A, Duan S, Kistner EO, Nicolae DL, et al SCAN: SNP and copy number annotation. Bioinformatics. 2010;26(2):259–62. PubMed Central PMCID: PMC2852202. 10.1093/bioinformatics/btp644 19933162 PMC2852202

[32] Benjamini Y, Hochberg Y. Controlling the false discovery rate: a practical and powerful approach to multiple testing. Journal of the Royal Statistical Society, Series B. 1995;57(1):289–300.

[33] Liu JZ, McRae AF, Nyholt DR, Medland SE, Wray NR, Brown KM, et al A versatile gene-based test for genome-wide association studies. American journal of human genetics. 2010;87(1):139–45. PubMed Central PMCID: PMC2896770. 10.1016/j.ajhg.2010.06.009 20598278 PMC2896770

[34] Schegg B, Hulsmeier AJ, Rutschmann C, Maag C, Hennet T. Core glycosylation of collagen is initiated by two beta(1-O)galactosyltransferases. Molecular and cellular biology. 2009;29(4):943–52. Epub 2008/12/17. PubMed Central PMCID: PMCPmc2643808. 10.1128/MCB.02085-07 19075007 PMC2643808

[35] van Gent RN, Siem D, van Middelkoop M, van Os AG, Bierma-Zeinstra SMA, Koes BW. Incidence and determinants of lower extremity running injuries in long distance runners: a systematic review. British journal of sports medicine. 2007;41(1473–0480 (Electronic)):469–80. 10.1136/bjsm.2006.033548 17473005 PMC2465455

[36] Moses B, Orchard J, Orchard J. Systematic review: Annual incidence of ACL injury and surgery in various populations. Res Sports Med. 2112;(1543–8635 (Electronic)):157–79. 10.1080/15438627.2012.680633 22742074

[37] Moller A, Astron M, Westlin N. Increasing incidence of Achilles tendon rupture. Acta orthopaedica Scandinavica. 1996;67(5):479–81. Epub 1996/10/01. 8948254 10.3109/17453679608996672

[38] Lantto I, Heikkinen J, Flinkkila T, Ohtonen P, Leppilahti J. Epidemiology of Achilles tendon ruptures: increasing incidence over a 33-year period. Scandinavian journal of medicine & science in sports. 2015;25(1600–0838 (Electronic)):e133–8. 24862178 10.1111/sms.12253

[39] Ganse B, Degens H, Drey M, Korhonen MT, McPhee J, Muller K, Johannes BW, et al Impact of age, performance and athletic event on injury rates in master athletics—first results from an ongoing prospective study. J Musculoskelet Neruonal Interact. 2014;14(1108–7161 (Print)):148–54. 24879018

[40] Prodromos CC, Han Y, Rogowski J, Rogowski J, Joyce B, Shi K. A meta-analysis of the incidence of anterior cruciate ligament tears as a function of gender, sport, and a knee injury-reduction regimen. Arthroscopy. 23(1526–3231 (Electronic)):1320–5. 10.1016/j.arthro.2007.07.003 18063176

[41] Panagiotou OA, Ioannidis JP. What should the genome-wide significance threshold be? Empirical replication of borderline genetic associations. (1464–3685 (Electronic)). 10.1093/ije/dyr178 22253303

[42] Broer L, Lill CM, Schuur M, Amin N, Roehr JT, Bertram L, Ioannidis JPA, et al. Distinguishing true from false positives in genomic studies: p values. (1573–7284 (Electronic)). 10.1007/s10654-012-9755-x 23371043

[43] Ioannidis JP, Tarone R, McLaughlin JK. The false-positive to false-negative ratio in epidemiologic studies. Epidemiology. 2011;22(4):450–6. 10.1097/EDE.0b013e31821b506e 21490505

[44] Siontis KC, Patsopoulos NA, Ioannidis JP. Replication of past candidate loci for common diseases and phenotypes in 100 genome-wide association studies. European journal of human genetics: EJHG. 2010;18(7):832–7. PubMed Central PMCID: PMC2987361. 10.1038/ejhg.2010.26 20234392 PMC2987361

