# Supplementary figures and images for: Genome-wide association screens for Achilles tendon and ACL tears and tendinopathy
Source: PLoS One. 2017 Mar 30;12(3):e0170422. doi: 10.1371/journal.pone.0170422 (PMC5373512; doi:10.1371/journal.pone.0170422)

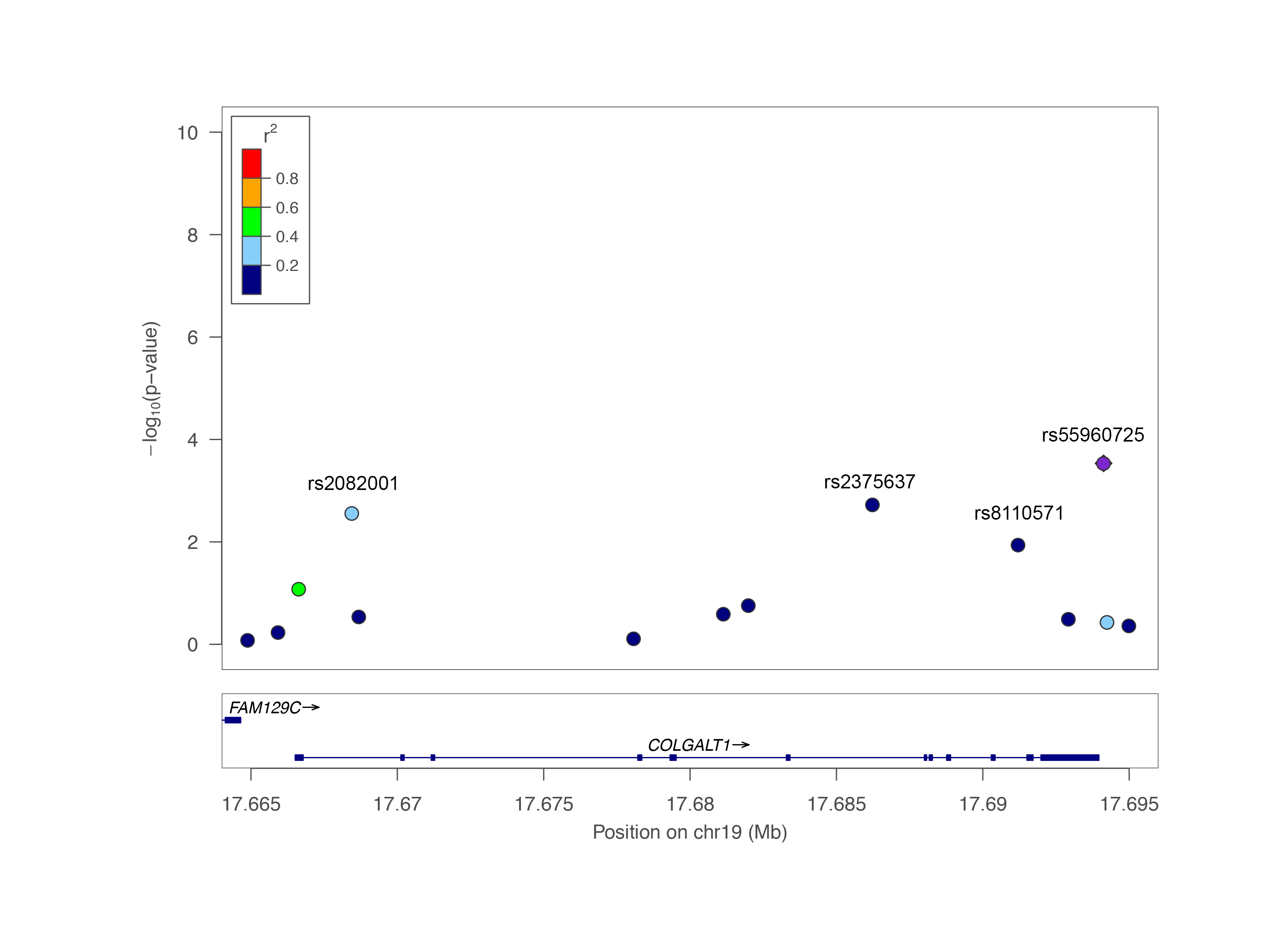

Supplement: S1 Fig — Tested SNPs within GLT25D1 are arranged by genomic position on chromosome 19 (x-axis). The y-axis indicates -log10 p-values for association with ACL rupture for each SNP. The four SNPs showing nominal statistical significance (p<0.05) are shown. The color of dots representing flanking SNPs indicates their linkage disequilibrium (r2) with the lead SNP as indicated in the heat map color key. (TIF) [file pone.0170422.s001.tif]
