# Supplementary material for: Genome-wide association screens for Achilles tendon and ACL tears and tendinopathy
Source: PLoS One. 2017 Mar 30;12(3):e0170422. doi: 10.1371/journal.pone.0170422 (PMC5373512; doi:10.1371/journal.pone.0170422)
Supplement: S1 Table — (DOCX) [file pone.0170422.s002.docx]

**S1 Table. Results from LD Score Regression.**

| **Phenotype** | **h^2^ (SE)^a^** | **Mean χ^2, b^** | **λ_GC_^c^** | **Intercept (SE)^d^** |
| --- | --- | --- | --- | --- |
| Achilles tendon inury | 0.0048 (0.011) | 1.01 | 1.03 | 1.00 (0.02) |
| ACL rupture | 0.011 (0.011) | 1.02 | 1.04 | 1.00 (0.02) |

^a^Heritability (standard error).

^b^Mean Chi-squared statistic.

^c^Genomic control.

^d^LD Score regression intercept (standard error).
