# Supplementary material for: Genome-wide association screens for Achilles tendon and ACL tears and tendinopathy
Source: PLoS One. 2017 Mar 30;12(3):e0170422. doi: 10.1371/journal.pone.0170422 (PMC5373512; doi:10.1371/journal.pone.0170422)
Supplement: S3 Table — (DOCX) [file pone.0170422.s004.docx]

**S3 Table. Re-testing candidate genes for association with Achilles tendon injury or ACL rupture without using co-variates.**

| **Achilles tendon injury** | |  |  |  |  |
| --- | --- | --- | --- | --- | --- |
| **SNP** | **Gene** | **EA^a^** | **P-value^b^** | **OR (95% CI)** | **Ref^c^** |
| rs1045485^d^ | CASP8 | C | 2.7x10^-3^ | 1.12 (1.05-1.19) | [2] |
| rs4747096 | ADAMTS14 | G | 0.12 | .97 (0.93-1.10) | [10] |
| rs2761884 | BMP4 | T | 0.50 | 1.02 (0.97-1.06) | [1] |
| rs1134170 | COL5A1 | A | 0.31 | .96 (0.88-1.04) | [5] |
| rs12722 | COL5A1 | T | 0.98 | 1.00 (0.92-1.08) | [4] |
| rs3196378 | COL5A1 | A | 0.92 | 1.00 (0.93-1.09) | [4] |
| rs1559186 | COL5A3 | C | 0.35 | 1.02 (0.98-1.07) | [6] |
| rs331079 | FBN2 | C | 0.48 | 1.02 (0.96-1.09) | [7] |
| rs4919510^e^ | MIR608 | G | 5.1x10^-3^ | .93 (0.89-0.98) | [5] |
| rs591058^d^ | MMP3 | T | 0.04 | 1.04 (1.00-1.08) | [9] |
| rs679620^d^ | MMP3 | T | 0.05 | 1.04 (1.00-1.08) | [9] |
| rs4789932^d^ | TIMP2 | G | 0.06 | 1.05 (1.00-1.11) | [10] |
| rs1330363 | TNC | C | 0.55 | 1.01 (0.97-1.05) | [12] |
| rs2104772 | TNC | A | 0.32 | 0.98 (0.94-1.02) | [12] |
| **ACL rupture** |  |  |  |  |  |
| **SNP** | **Gene** | **EA^a^** | **P-value^b^** | **OR (95% CI)** | **Ref^c^** |
| rs1516797 | ACAN | T | 0.95 | 0.99 (0.77-1.28) | [13] |
| rs516115 | DCN | A | 0.29 | 1.08 (0.94-1.24) | [13] |
| rs970547 | COL12A1 | T | 0.38 | 1.07 (0.92-1.24) | [19] |
| rs2276109 | MMP12 | T | 0.35 | 1.09 (0.91-1.31) | [20] |
| rs1800255^f^ | COL3A1 | A | 0.03 | 1.16 (1.01-1.34) | [21] |
| rs331079 | FBN2 | G | 0.79 | 1.03 (0.84-1.25) | [7] |
| rs679620 | MMP3 | T | 0.57 | 1.05 (0.93-1.19) | [22] |

^a^ Effect allele.

^b^ P-value from this study.

^c^ Reference showing original association of candidate SNP.

^d^ For rs1045485, rs591058, rs679620 and rs4789932, the direction of the effect was opposite to the previously published results [2][9][10].

^e^ For rs4919510, the direction of effect is the same as the previously published result [5].

^f^ For rs1800255, the direction of effect is the same as the previously published result [21].
