# Supplementary material for: Genome-wide association screens for Achilles tendon and ACL tears and tendinopathy
Source: PLoS One. 2017 Mar 30;12(3):e0170422. doi: 10.1371/journal.pone.0170422 (PMC5373512; doi:10.1371/journal.pone.0170422)
Supplement: S4 Table — (DOCX) [file pone.0170422.s005.docx]

**S4 Table. List of 90 Candidate Genes**

| Protein | Location/Function | Genes |
| --- | --- | --- |
| Type I collagen | extracellular matrix | COL1A1 |
|  |  | COL1A2 |
| Type II collagen | extracellular matrix | COL2A1 |
| Type III collagen | extracellular matrix | COL3A1 |
| Type IV collagen | basement membrane | COL4A1 |
|  |  | COL4A2 |
|  |  | COL4A3 |
|  |  | COL4A4 |
|  |  | COL4A5 |
| Type V collagen | extracellular matrix | COL5A1 |
|  |  | COL5A2 |
|  |  | COL5A3 |
| Type VI collagen | extracellular matrix - distal and proximal thirds | COL6A1 |
|  |  | COL6A2 |
|  |  | COL6A3 |
| Type XI collagen | extracellular matrix | COL11A1 |
|  |  | COL11A2 |
| Type XII collagen | extracellular matrix | COL12A1 |
| Type XIV collagen | extracellular matrix | COL14A1 |
| Type XVIII/Endostatin | affects extracellular matrix | COL18A1 |
| Versican | extracellular matrix | VCAN |
| Aggrecan | articular cartilage | ACAN |
| Biglycan | extracellular matrix | BGN |
| Fibromodulin | extracellular matrix | FMOD |
| Lumican | extracellular matrix | LUM |
| PREL protein | extracellular matrix | PRELP |
| Epiphycan | extracellular matrix | EPYC |
| Perlecan | basement membrane | HSPG2 |
| Keratocan | extracellular matrix | KERA |
| Agrin | basement membrane | AGRN |
| Decorin | extracellular matrix | DCN |
| Cartilage oligomeric extracellular matrix protein | extracellular matrix | COMP |
| Elastin | extracellular matrix | ELN |
| Actin alpha 2 | extracellular matrix | ACTA2 |
| Laminin | basement membrane | LAMA1 |
|  |  | LAMA2 |
|  |  | LAMA3 |
|  |  | LAMA4 |
|  |  | LAMA5 |
|  |  | LAMB1 |
|  |  | LAMB2 |
|  |  | LAMB3 |
|  |  | LAMB4 |
|  |  | LAMC1 |
|  |  | LAMC2 |
|  |  | LAMC3 |
| Integrin | extracellular matrix | ITGA5 |
|  |  | ITGA2B |
|  |  | ITGB1 |
|  |  | ITGA6 |
|  |  | ITAGV |
| Fibrillin | extracellular matrix | FBN1 |
|  |  | FBN2 |
| Tenascin-C | extracellular matrix | TNC |
| Entactin | basement membrane | NID1 |
| Insoluble cellular fibronectin | extracellular matrix | FN1 |
|  |  | FSD1 |
|  |  | FNDC4 |
| Extracellular matrix metalloproteinase | degrades collagen and other matrix components | MMP1 |
|  |  | MMP2 |
|  |  | MMP3 |
|  |  | MMP7 |
|  |  | MMP8 |
|  |  | MMP9 |
|  |  | MMP10 |
|  |  | MMP12 |
|  |  | MMP13 |
|  |  | MMP14 |
|  |  | MMP15 |
|  |  | MMP16 |
|  |  | MMP17 |
|  |  | MMP18 |
|  |  | MMP19 |
|  |  | MMP25 |
|  |  | MMP26 |
| Cathepsin B | protease | CTSB |
| Transcription factor Sox-9 | chondrocyte differentiation | SOX9 |
| Procollagen galactosyltransferase 1 | modifies collagen | GLT25D1 |
| Lysyl hydroxylase 1 | modifies collagen | PLOD1 |
| Lysyl hydroxylase 2 | modifies collagen | PLOD2 |
| Lysyl hydroxylase 3 | modifies collagen | PLOD3 |
| Galactosylhydroxylysyl glucosyltransferase | modifies collagen | GGT |
| Lysyl oxidase | promotes crosslink formation | LOXL1 |
| Peptidyl proline *cis-trans* isomerase B | catalyzes isomerization of peptide bonds | PPIB |
| Disulphide isomerase | collagen synthesis | PDIA2 |
|  |  | PDIA3 |
|  |  | PDIA4 |
|  |  | PDIA5 |
|  |  | PDIA6 |
| Heat shock protein 47 | molecular chaperone | SERPINH1 |
