# Supplementary material for: Genome-wide association screens for Achilles tendon and ACL tears and tendinopathy
Source: PLoS One. 2017 Mar 30;12(3):e0170422. doi: 10.1371/journal.pone.0170422 (PMC5373512; doi:10.1371/journal.pone.0170422)
Supplement: S7 Table — (DOCX) [file pone.0170422.s008.docx]

**S7 Table. Association of SNPs in *GLT25D1* with ACL rupture.**

| SNP | EA^a^ | r^2^ to rs55960725^b^ | P meta^c^ | OR meta (95% CI)^d^ | P cond.^e^ | OR cond. (95% CI)^f^ |
| --- | --- | --- | --- | --- | --- | --- |
| rs2082001 | T | 0.21 | 2.7x10^-3^ | 1.26 (1.11-1.41) | 0.15 | 1.14 (0.91-1.65) |
| rs2375637 | C | 0.19 | 1.9x10^-3^ | 0.65 (0.38-0.92) | 0.10 | 0.77 (0.96-1.32) |
| rs8110571 | G | 0.08 | 1.1x10^-2^ | 1.28 (0.91-1.65) | 0.63 | 1.11 (0.69-1.53) |
| rs55960725 | T | NA | 2.9x10^-4^ | 0.78 (0.65-0.91) | NA | NA |

^a^Effect allele.

^b^ Correlation to rs55960725.

^c^P-value from fixed effects meta-analysis.

^d^Allelic odds ratio from fixed effects meta-analysis (95% confidence interval).

^e^Conditional p-value from fixed effects meta-analysis using rs55960725 as a covariate.

^f^Odds ratio using rs55960725 as a covariate (95% confidence interval).
